# Is Primary Chancre Ever a Derivative of Secondary Syphilis by Inoculation?

**Published:** 1861-08

**Authors:** 


### IS PRIMARY CHANCRE EVER A DERIVATIVE OF SECONDARY SYPHILIS BY INOCULATION?

---

*Messrs. Editors:*

Having recently under my observation a case, whose history seems to afford an affirmative reply to this grave question, I propose to give a brief outline of it to your readers, hoping that such an account may elicit corroborative facts from the experience of my professional brethren.

Early in April last, the patient, a young lady, in excellent health at the time, noticed a "cold sore" upon her under lip. It presented no unusual appearance and no application was made to it until about ten days from its commencement, when its indisposition to heal, and the swelling at its base gave rise to some degree of apprehension on the part of the patient. About the same time the cervical glands began to enlarge and were painful, the mucous membrane of the fauces was red and "raw," and the patient was unable after a time to swallow any other than the blandest fluids. The lower lip became largely swollen and everted, the base of the sore extensively indurated—its surface secreting in small quantities a thin unhealthily appearing pus. No local applications of which every variety was tried, were of the slightest avail toward ameliorating the condition either of the lip or of the throat.

In May, her state was truly deplorable, the surface of the body being here and there studded with an eruption, dry and scaly, and her general appearance indicating a deeply seated cachexia. During the second week in May she began to take the Proto Iodide of Mercury, and from that time her improvement was continuous and rapid.

By the 1st of June—not quite three weeks from the first administration of the mercurial, the lip was entirely restored, but a slightly marked cicatrix remaining and that not perceptibly indurated. Her throat also was well, the cervical glands reduced to their natural size and the skin clear and free from any eruption. She then took Iron in Huxham's tincture for several weeks with benefit to her general health and strength.

About the 1st of August, she noticed a slight renewal of the eruption, upon the face, scalp and extremities, but this vanished again under the use of the Bichloride of Mercury.

Few professional men, I think, would have, under these circumstances, entertained a doubt as to the specific character of the disease. The indurated base of the disease, the affection of the throat and skin, the well-marked cachexia, and finally the immediate and curative influence of the mercurials seem to furnish indubitable evidence to the correctness of my assumption.

In the course of my painful investigation with reference to the origin of the evil, I ascertained that the patient was engaged to be married to a young gentleman, who was and is now suffering from secondary Syphilis, his throat having been for a period of six months, from time to time the *locale* of extensive and characteristic ulcerations.

The evidence and testimony in the case, not to particularize, are convincing to my mind, that none other than the most proper and customary intercourse has ever existed between these parties, and accordingly I fully believe that the patient, by the contact alone of lip with lip, has received into her system the syphilitic virus—in short, that from the products of secondary ulceration a primary chancre in this case resulted by inoculation.

"AB UNO, DISCE OMNES."

*Remarks.*—The above was received too late for insertion under the appropriate head. Aside from the question which must ever remain undecided, save to the minds of those who have the immediate personal cognizance of such cases and have necessarily individual "generous confidence," there arises the query whether the inoculation might not after all have been of *primary* syphilitic virus. The modes in which this might occur will suggest themselves to any one who has observed a case of gonnorrhœal ophthalmia. Again primary syphilitic ulceration of the throat is a condition which must be recognized as occasional, and as well established as the occurrence of bubo.

The important interrogatory proposed by our correspondent can only be solved by the production of primary chancre by direct inoculation with the products of secondary syphilitic ulceration. Thus far we have no confidence in the result of experiments.

In the particular case above given the acute observer who furnishes the record, we have every reason to believe, could not have been mistaken as to the nature of the disease of the lip.

*Lectures on the Diagnosis and Treatment of the Principal Forms of Paralysis of the Lower Extremities.*—By E. Brown Sequard, M. D., F. R. S., &c. Pp. 118. Price $1.50.

*Course of Lectures on the Physiology and Pathology of the Central Nervous System.*—By the same. Pp. 276, with plates appended. Price $2.25.—Both from the Publishers, J. B. Lippincott & Co., Philadelphia, per S. C. Griggs & Co., Lake street, Chicago.

The pressure of other duties upon our time since the reception of the above works, has prevented our noticing them in detail, as we intend speedily to do. Meanwhile, in announcing them to our readers, we feel confident that they will hail their appearance with great pleasure. No physician who wishes to know anything about the nervous system, in health or disease, should fail to secure them at once. They are invaluable. In whatever particular individuals may differ from the author's conclusions, the perfection of his experiments and observations must everywhere be conceded. In this particular department of physiology, Brown Sequard stands second to no living observer, and certainly he describes what he has seen with a clearness and precision which renders it a rich treat to read his writings. But "more anon."

---

*Graduate of Rush Med. College.*—The name of W. Bates, M. D., of Ransom Centre, Mich., was inadvertently omitted from the list of graduates of Rush Med. College in the March number of this Journal and the Annual Announcement. Dr. Bates is a gentleman of fine scholarship and an honor to any institution as an alumnus.

---

*Medical Director.*—Dr. J. K. Barnes has been attached to the staff of Maj. Gen. Hunter as Medical Director. It is an appointment eminently fit to be made, and will inspire general confidence in the medical corps of the army of the Northwest.

